# Dynamically rebalancing the unstable shoulder in Ehlers-Danlos syndrome: latissimus dorsi transfer for posterior shoulder instability

**DOI:** 10.1016/j.jseint.2024.05.008

**Published:** 2024-05-28

**Authors:** Sameer R. Khawaja, Zaamin B. Hussain, Hayden Cooke, Elinor Stern, Anthony Karzon, Michael B. Gottschalk, Eric R. Wagner

**Affiliations:** a Department of Orthopaedic Surgery, Emory University School of Medicine, Atlanta, GA, USA; b Department of Orthopaedic Surgery, University of Michigan School of Medicine, Ann Arbor, MI, USA

**Keywords:** Posterior instability, Ehlers-Danlos syndrome, Scapular dyskinesia, Tendon transfer, Digital dynamic radiography, Multidirectional instability, Shoulder instability, Latissimus transfer

Ehlers-Danlos syndrome (EDS) is a family of connective tissue disorders resulting from a collagen formation defect. The prevalence of EDS is between 0.2/1000 and 10/1000 people, with the hypermobile type accounting for up to 80%-90% of EDS patients.^47^ The most common manifestations of hypermobile EDS (hEDS) include generalized ligamentous hyperlaxity, recurrent symptomatic subluxations/dislocations, tendon ruptures, chronic musculoskeletal pain (including fibromyalgia), and functional impairment.^47^^,^^48^ hEDS can be described by its 3 chronological phases: the “hypermobility” phase, “pain” phase that begins during adolescence, and “stiffness” phase, appearing in adulthood and characterized by progressive limitations in joint motion.^15^ Joint hypermobility can be quantified using the Beighton score (Table 1).^3^^,^^8^^,^^20^ The shoulder is among the most commonly affected joints,^16^^,^^42^ due to its reliance on static and dynamic soft tissue stabilizers and lack of inherent bony stability. This also makes management challenging, as stabilization of pathologic recurrent shoulder instability with lax soft tissues increases the risk of recurrence.

**Table I:** Beighton score for joint hypermobility.

Beighton scale	Left	Right
Passive dorsiflexion and hyperextension of 5th MCP joint beyond 90°	1	1
Passive apposition of the thumb to flexor aspect of forearm	1	1
Passive hyperextension of elbow beyond 10°	1	1
Passive hyperextension of knee beyond 10°	1	1
Active forward flexion of trunk with knees fully extended so palms of hands rest flat	1	
Total	/9	

*MCP*, metacarpophalangeal joint.

Although posterior shoulder instability accounts for <10% of all cases of shoulder instability,^5^^,^^10^^,^^22^ patients with hEDS are predisposed to both posterior and multidirectional shoulder instability.^32^^,^^36^^,^^42^ While the failure rate of arthroscopic capsulorrhaphy and posterior labral repairs is less than 10% in patients without hEDS,^10^ these procedures are not as effective in EDS where the tissue structural integrity is poor.^32^ When these procedures fail, the patient is left with limited options.^50^ The joint preservation options include arthroscopic or open posterior bone blocks,^14^^,^^19^ capsular reconstructions with allograft,^29^ and glenoid osteotomy if there is concomitant severe glenoid retroversion,^55^ with salvage reconstruction options such as arthrodesis or arthroplasty as a last resort.^5^^,^^50^

Rebalancing the dynamic soft tissue stabilizers and deforming forces in patients with hEDS may represent an alternative approach. The latissimus dorsi (LD) originates on the posterior aspect of the hemithorax and inserts anteriorly in the floor of the bicipital groove. This functions not only as a humerothoracic internal rotator and adductor but it also pulls the humeral head posteriorly and inferiorly, making it a significant contributor to posterior instability when overactive.^40^ Although LD tendon transfer has been described extensively for irreparable rotator cuff tears,^4^^,^^13^^,^^25^^,^^38^^,^^43^^,^^54^^,^^58^ its use to treat posterior shoulder instability has not been reported. Furthermore, many patients with hEDS have scapular dyskinesia due to pectoralis minor (PM) overactivity, leading to an increased risk of instability, particularly in the posterior direction.^12^^,^^52^^,^^53^ We present a novel approach to surgical management of recurrent posterior shoulder instability in hEDS using an arthroscopically assisted LD transfer with an arthroscopic PM release, and confirm reduction radiographically with dynamic digital radiography.

## Case report

### Patient presentation and physical examination

A 14-year-old female with hEDS presented with a 5-year history of recurrent atraumatic left shoulder dislocations and subluxations. Her past medical history was significant for patellar and hip instability and contralateral right shoulder posterior instability treated twice with arthroscopic capsulorrhaphies. On the left, initially, an arthroscopic posterior 3-anchor capsulorrhaphy procedure with nonabsorbable sutures had been performed 3 years prior and failed with multiple subsequent posterior instability events. She subsequently underwent a revision arthroscopic anterior and posterior capsular plication and rotator interval closure. This then failed on advancing motion out of the immobilizer, with a recurrent posterior dislocation event. She underwent multiple, unsuccessful courses of physical therapy focused on strengthening and retraining her rotator cuff and periscapular musculature. She reported more than 100 dislocations or severe subluxations, had received more than 8 corticosteroid injections into her glenohumeral joint, and was on chronic oral opioids for the preceding 3 years.

**Figure 1:**
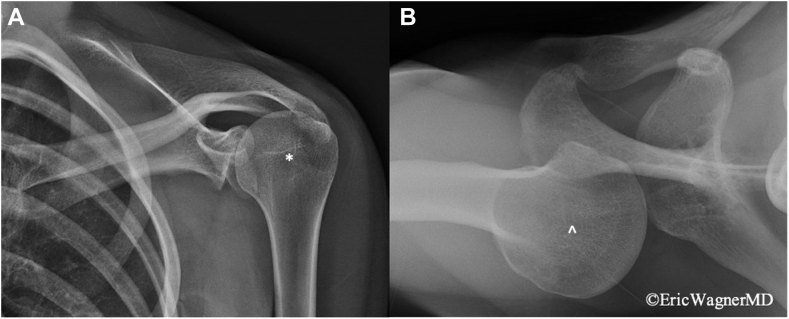
Grashey (**A**) and axial (**B**) radiographs of left shoulder. The glenohumeral joint space is poorly visualized on the Grashey view, and the humeral head is superiorly migrated. Axial view demonstrates posterior subluxation of the humeral head, and the glenoid boundaries are challenging to define.

**Figure 2:**
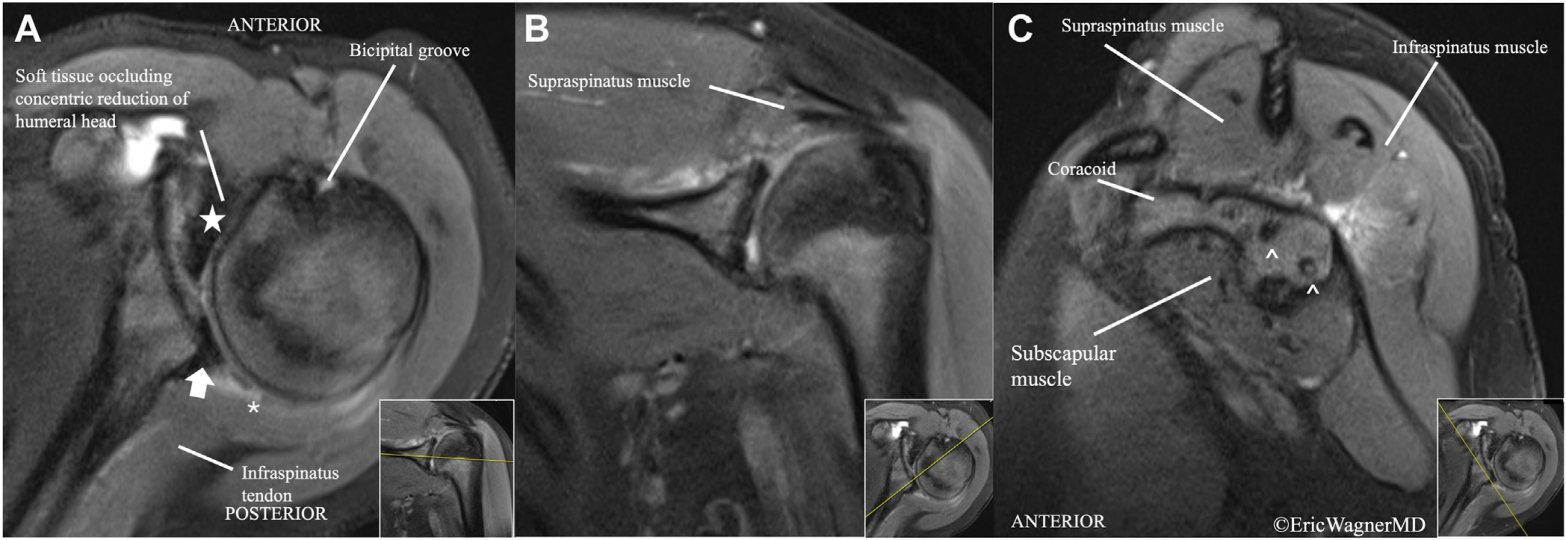
Proton density fat-saturated magnetic resonance imaging of left shoulder in axial (**A**), coronal (**B**), and sagittal (**C**) planes. (**A**) Axial view demonstrates severe glenoid dysplasia and retroversion with posterior capsulolabral complex detachment and soft tissue interposition in the anterior joint (*star*). Chondral defect at humeral head (∗). (**B**) Coronal view demonstrates articular defects in location of previous anchor tracks. (**C**) Sagittal view demonstrates postsurgical anchor tracks in the glenoid from previous capsulolabral fixation (ˆ), and a deficient glenoid.

**Figure 3:**
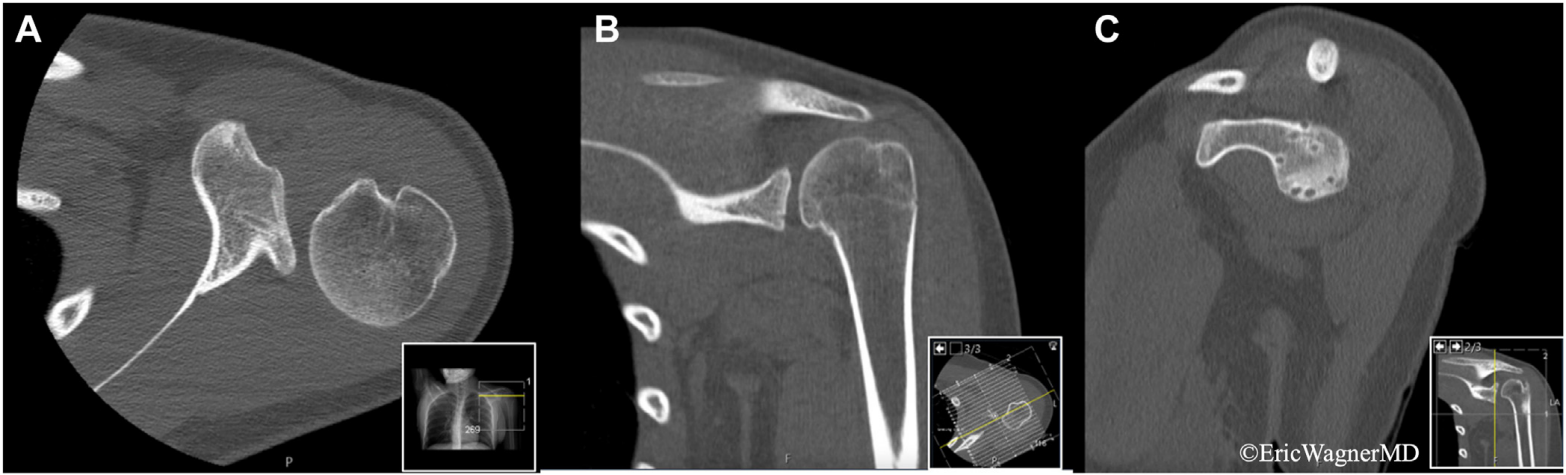
Computed tomography (CT) of left shoulder in axial (**A**), coronal (**B**), and sagittal (**C**) planes. Axial view demonstrates posterior subluxation of shoulder. Sagittal view demonstrates multiple glenoid cortical defects representing postsurgical anchor tracks from previous capsulolabral fixation.

**Figure 4:**
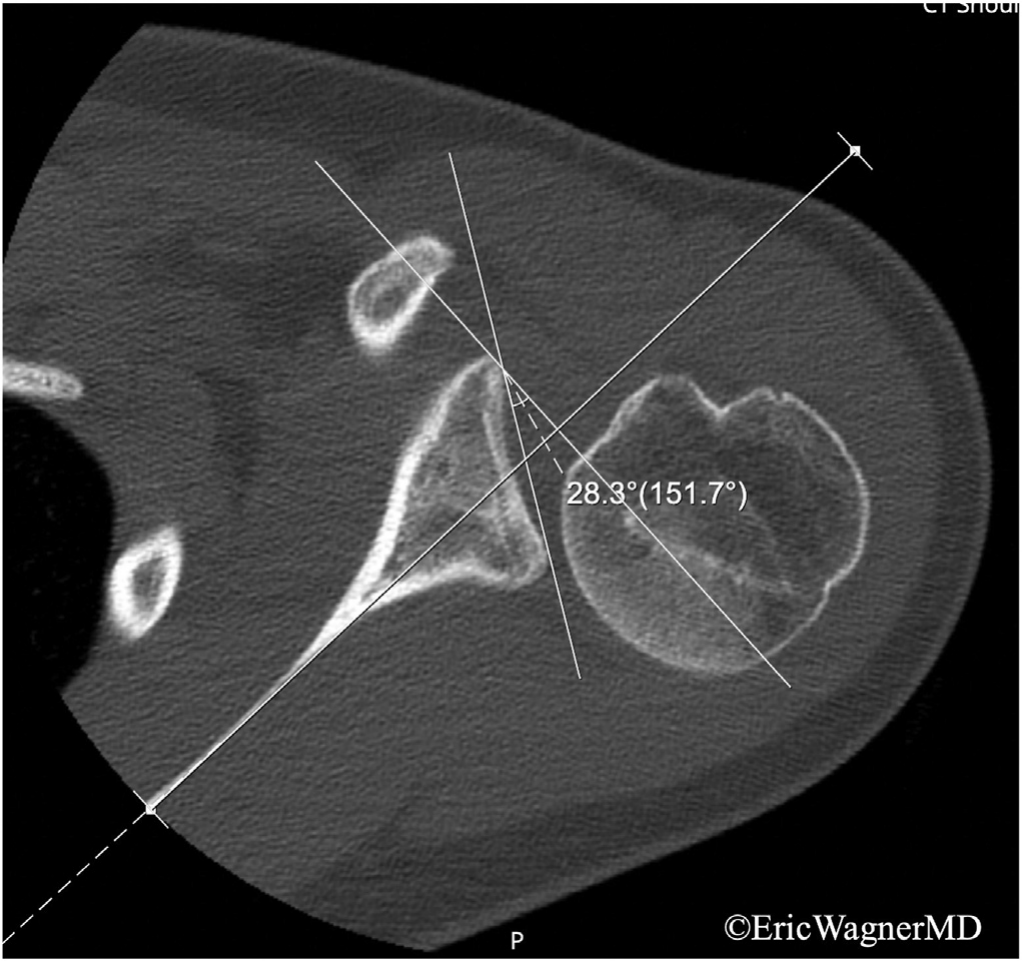
Axial CT image of left glenohumeral joint displaying Walch type B3 dysplastic glenoid with 28.3° of retroversion, and significant posterior humeral head subluxation in the setting of capsulolabral hyperlaxity. *CT*, computed tomography.

**Figure 5:**
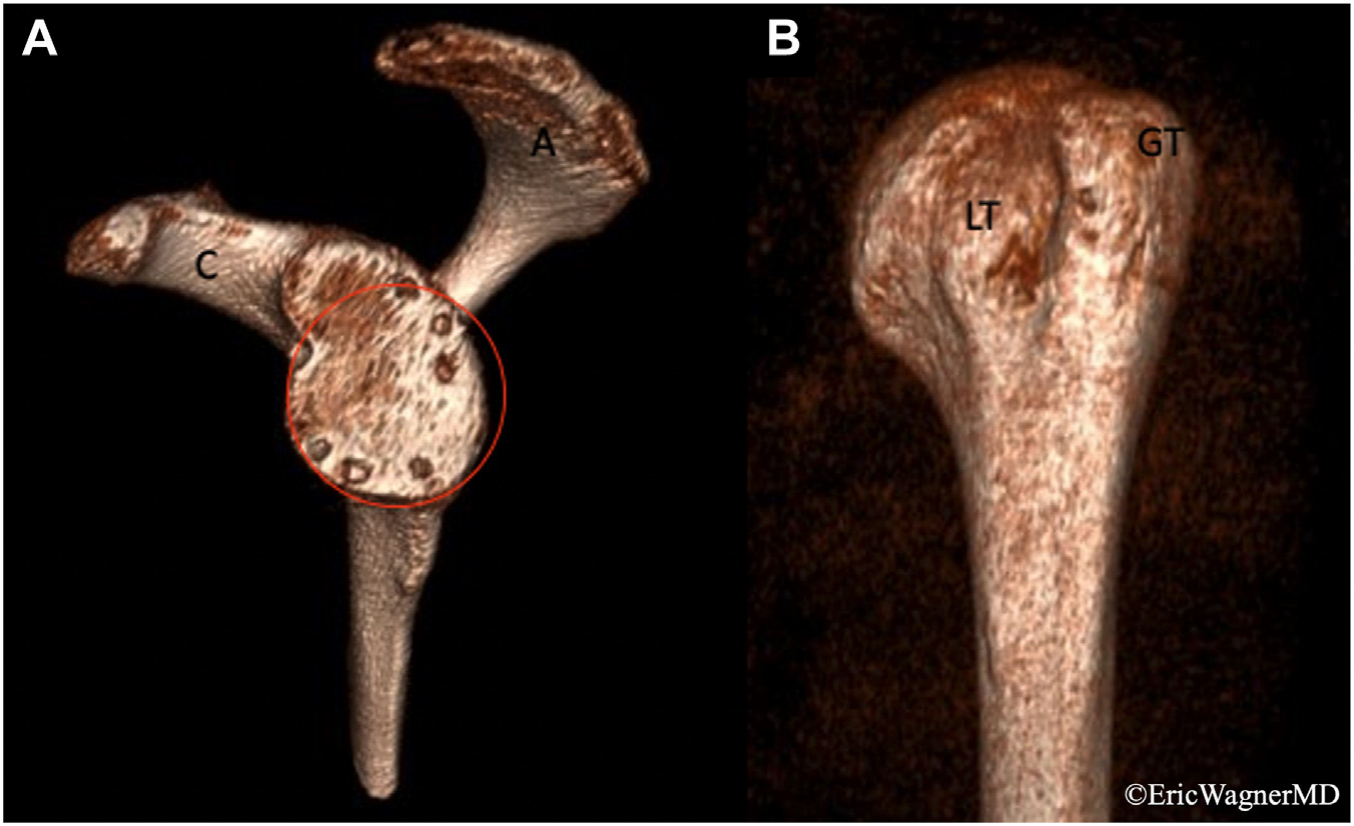
Three-dimensional reconstruction of left scapula and glenoid en face (**A**) and proximal humerus (**B**). Posterior glenoid bone loss is seen when best-circle method applied. Coracoid (C), acromion (A), lesser tuberosity (LT), greater tuberosity (GT).

**Figure 6:**
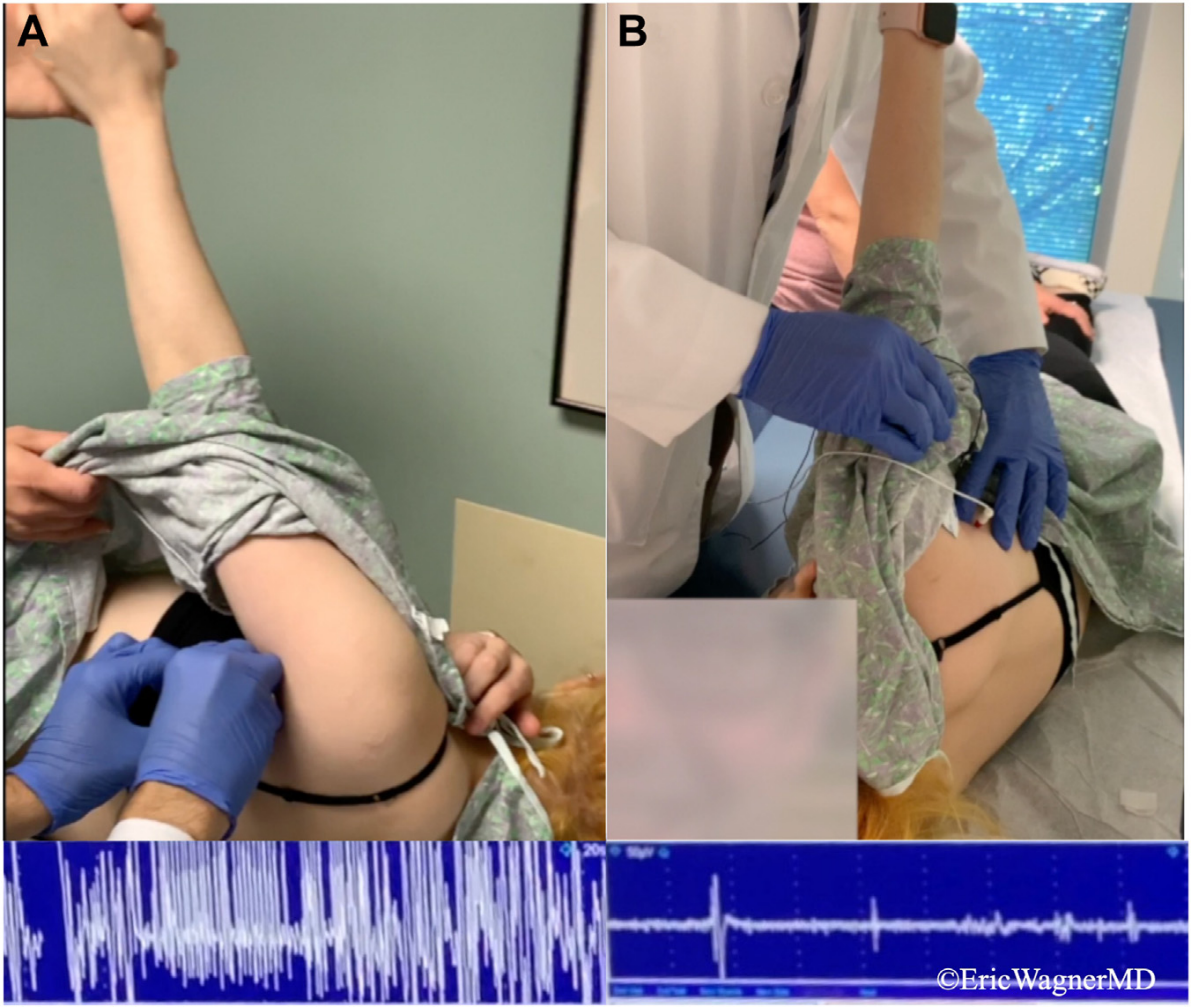
A dynamic electromyogram (EMG) was performed, as latissimus dorsi spasming was encountered on clinical examination. On the side of interest, abnormal rapid firing patterns were recorded (**A**), compared to the contralateral latissimus dorsi (**B**).

Upon her initial consultation, she reported pain with most motions of her shoulder, with limitations in active range of motion in flexion (90°), abduction (70°), external rotation (40°), and internal rotation (side) secondary to pain. Her Visual Analogue Scale pain was rated at 8/10, shoulder Single Assessment Numeric Evaluation (SANE) score was 5%, and American Shoulder and Elbow Surgeons (ASES) score was 10. On examination, she had multidirectional instability with a subtle sulcus sign. Her Beighton score was 9. She had positive posterior provocative maneuvers, including the Kim^54^ and Jerk^56^ tests, with grade 3 posterior subluxation of her humeral head relative to the glenoid. She had a negative anterior relocation test and only grade 1 anterior subluxation. Furthermore, she had significant LD spasms at rest and with attempted elevation or abduction. She was also tender to palpation at the PM insertion on to the coracoid, with a positive Tinel’s sign under it, and had marked scapular dyskinesia associated with a resting protracted posture of her scapula. Her PM length was 12 cm on the involved side compared to 14 cm on the contralateral side. Radiographs demonstrated severe posterior humeral head subluxation on the axillary view (Fig. 1), while magnetic resonance imaging showed a severely retroverted and dysplastic glenoid with posterior capsulolabral complex redundancy and detachment, and a reverse Hill-Sachs lesion (Fig. 2). Furthermore, computed tomography demonstrated glenoid retroversion of 28.3° using Friedman method,^28^ with associated posterior subluxation of the humeral head relative to the glenoid (Figs. 3 and 4). Three-dimensional reconstruction of the shoulder joint revealed posterior glenoid bone loss (Fig. 5). Given the LD spasms on examination, a dynamic electromyogram was performed that demonstrated abnormal LD firing at rest and humerothoracic abduction (Fig. 6). Given these findings, she was referred for botulinum toxin injection to the LD muscle, significantly improving her shoulder pain and subluxations for around 6 weeks.

**Figure 7:**
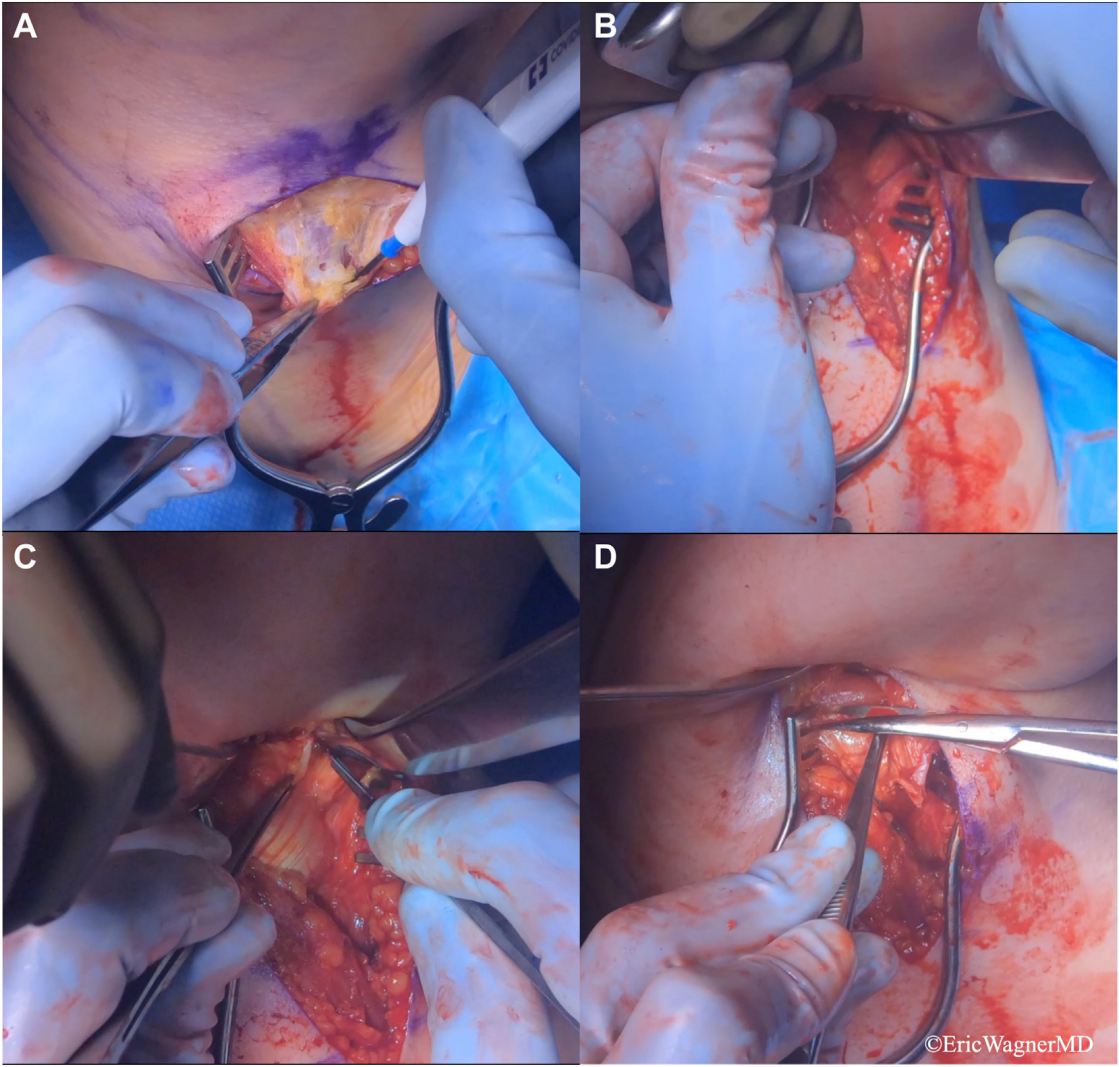
Harvest of the latissimus dorsi (LD). (**A**) A linear incision is made in the posterior axillary fold, and the soft tissue dissection is carried down through the fascia. (**B**) The muscle belly of teres major is first encountered. (**C**) The stripy white fibers of the LD tendon are identified. The tendon is followed anteriorly to the insertion on the humerus. The tendon is sharply excised from its insertion on the humerus. (**D**) The LD tendon is carefully separated from the teres major muscle belly.

**Figure 8:**
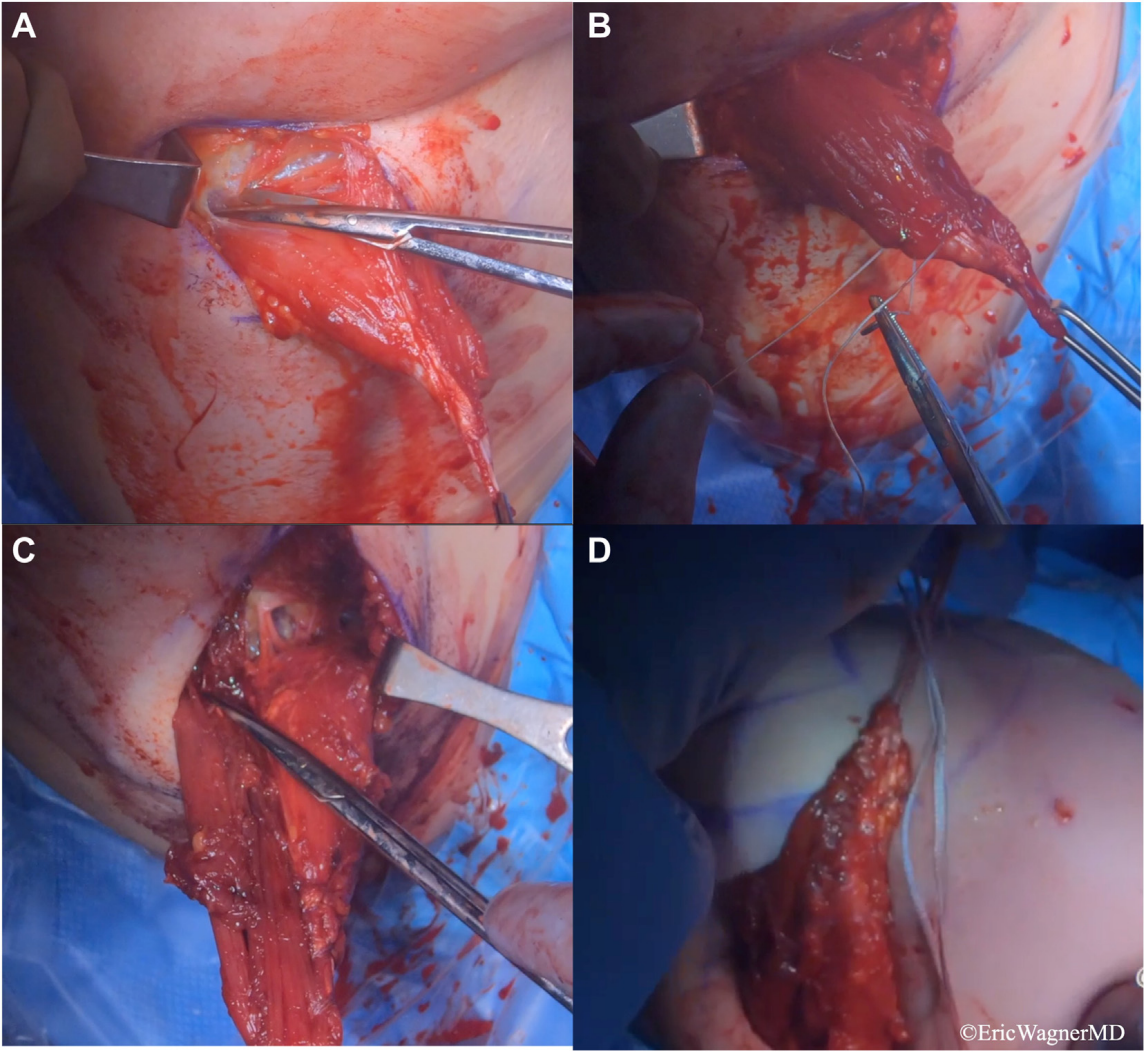
Mobilization and preparation of the latissimus dorsi (LD) tendon. (**A**) Adhesions are released from the LD tendon and muscle belly proximally. (**B**) The tendon is prepared for transfer with krackow sutures and a running baseball stitch. (**C**) Proximal superficial and deep adhesions are released to mobilize the LD tendon further. (**D**) Adequate tendon excursion without allograft augmentation is confirmed.

**Figure 9:**
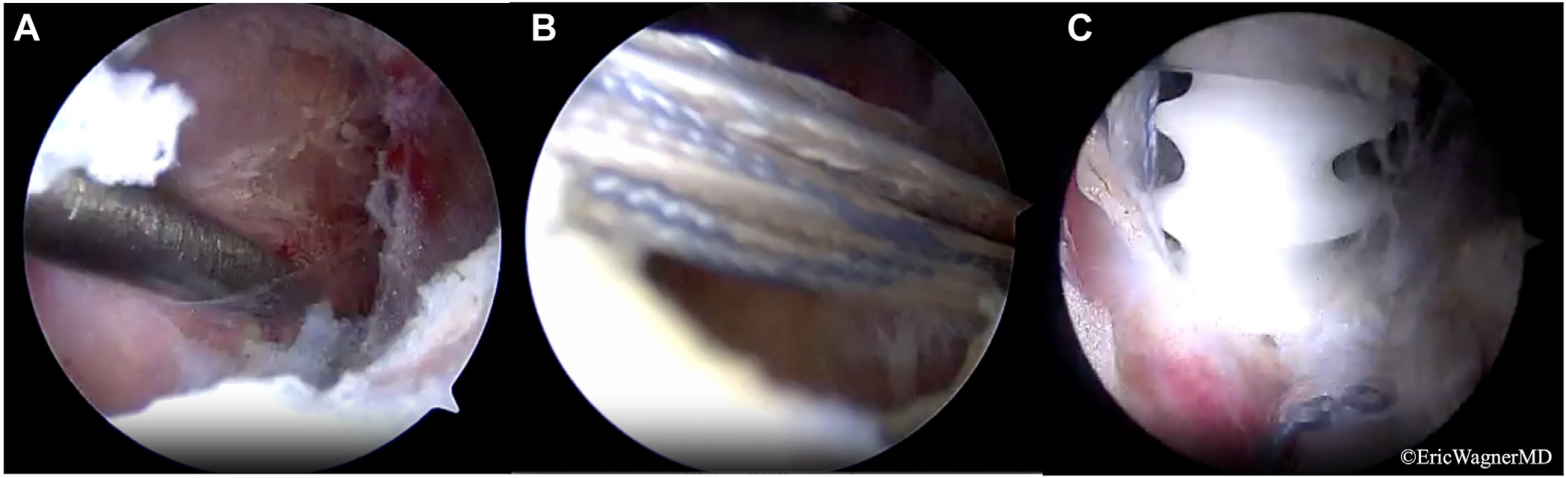
Arthroscopic portion of the transfer. (**A**) An interval for transfer is developed over the infraspinatus and teres minor muscles with the assistance of a dilated foley catheter which is slid back and forth. (**B**) The tendon sutures are advanced toward the lateral aspect of the greater tuberosity. (**C**) Two suture anchors placed lateral to the bicipital groove and tuberosity secure the transfer.

Given the reemergence of her scapular dyskinesia and posterior shoulder instability events after botulinum injection wore off, a decision was made to proceed with surgical intervention. The patient underwent an arthroscopic-assisted LD tendon transfer from its insertion anteriorly in the floor of the bicipital groove, to cover the shoulder posteriorly and insert at the lateral aspect of the greater tuberosity (GT) (Figure 7, Figure 8, Figure 9). This was intended to dynamically correct the posterior shoulder instability and direct the force vector from posterior to anterior, enabling external rotation and blocking posterior translation. Furthermore, an arthroscopic PM release was performed to correct the scapular dyskinesia and help rebalance the shoulder instability.

### Surgical technique

Using standard portals and beach chair positioning, a subacromial decompression was performed. Using techniques previously described,^1^^,^^2^^,^^46^^,^^51^ the PM was exposed and completely released from the coracoid, showing immediate retraction, signifying its overactivity and contribution to the scapular dyskinesia.

Using an anterolateral viewing portal and anteromedial working portal, the insertion of the LD was exposed. The tendon was sharply released and mobilized from the teres major. Next, an open incision was performed using a longitudinal incision in the posterior axillary fold along the anterior aspect of the LD musculotendinous unit (Fig. 7*A*). The LD tendon was dissected away from the teres major muscle (Fig. 7*B*, *C*, and *D*). The muscle was released to the level of the scapular inferior border, enabling it to be mobilized up to the superior aspect of the acromion. A Krakow stitch followed by a running baseball stitch was performed to create 2 sutures on each end of the tendon (Fig. 8*B*). Arthroscopically, a motorized shaver was used to prepare an area on the GT for the latissimus transfer.

The plane was developed arthroscopically between the residual infraspinatus and teres minor (Fig. 9*A*). A long hip arthroscopy grasper was passed from the anterolateral portal through the back incision. The sutures at the end of the tendon were retrieved and the tendon was pulled onto the lateral part of the GT (Fig. 9*B*). The tendon was fixed with 2 anchors on the lateral GT just lateral and superior to the teres major insertion (Fig. 9*C*). Postoperative rehabilitation protocol included 6 weeks in a sling, followed by physical therapy focusing on scapulohumeral retraining and strengthening beginning at 12 weeks.

### Postoperative course

**Figure 10:**
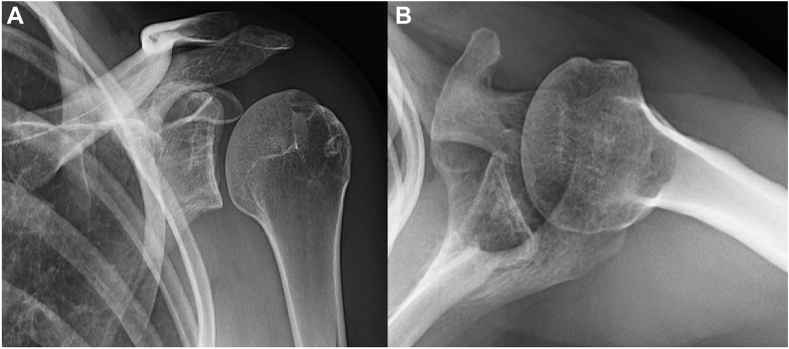
Grashey (**A**) and axial (**B**) radiographs of left shoulder at 31 months of follow-up. Both views demonstrate concentric reduction of the shoulder.

**Figure 11:**
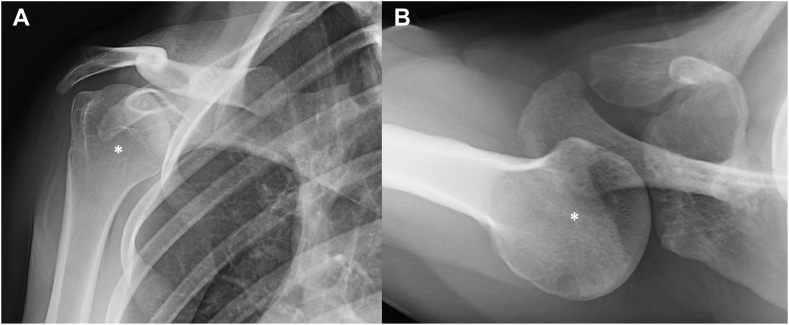
Grashey (**A**) and axial (**B**) radiographs of the contralateral right shoulder at 24 months of follow-up. Both views demonstrate a concerning posterior subluxation of the shoulder with glenoid retroversion, similar to the initial presentation of the left side.

The patient recovered very well postoperatively, with Visual Analogue Scale pain score of 3/10 at 6 weeks and 0/10 at 3, 7, 11, and 24 months postoperatively. Her shoulder SANE score improved to 50% at 6 weeks, 80% at months, and 90% at 7, 11, and 24 months postoperatively. At 7, 11, and 24 months, her ASES scores were 75, 82, and 85, respectively. At 2 months, she had full abduction (150°), forward flexion (170°), external rotation (70°), and internal rotation (>T10). She has had no subsequent dislocation or subluxation events on that shoulder. At her most recent follow-up at 2.9 years, she reported no issues with this shoulder and has regained complete motion without pain, with a SANE score of 90% and ASES of 88. A point of care ultrasound at 6 months, 1 year, and 2.9 years postoperatively performed in the clinic showed a completely healed LD following transfer. On static radiographs (Fig. 10) of the surgical shoulder, the patient had a well-centered humeral head without any signs of posterior subluxation. Dynamic radiography (Video 1), which has been shown to have utility in diagnosing dynamic shoulder pathology,^33^^,^^37^^,^^46^^,^^59^ demonstrated humeral head concentric relocation and stability, facilitating glenohumeral motion.^33^ She eventually started having symptoms on the contralateral shoulder static radiographs, dynamic images (Fig. 11, Video 1), and computed tomography scan demonstrated posterior subluxation associated with glenoid dysplasia and retroversion.

## Discussion

hEDS is a common subtype of EDS, characterized by more than 95% of patients experiencing frequent instability across all joints, often requiring surgical management.^12^^,^^47^ The reliance on dynamic and static soft tissue stabilizers without the bony stability within the glenohumeral joint leaves patients at risk of posterior dislocation and subluxation, especially in hEDS.^12^ When primary arthroscopic stabilization procedures fail to maintain shoulder stability, bone block procedures, arthroplasty, and arthrodesis are potential salvage procedures.^5^^,^^12^^,^^22^^,^^42^ In this particular case, the patient exhibited recalcitrant shoulder instability after she underwent multiple failed stabilization procedures, including 2 arthroscopic capsulorrhaphies, a capsular plication, and a rotator interval closure. She continued to experience pain and posterior humeral head dislocations with PM overactivity leading to marked scapular dyskinesia and a resting protracted posture of the scapula. Following a positive response to LD Botox injections, this case demonstrates the novel application of an arthroscopic-assisted LD tendon transfer for recurrent posterior shoulder instability in patients diagnosed with hEDS.

Traditionally, hEDS treatment begins with physical therapy and activity modification, followed by surgery.^11^^,^^12^^,^^48^ There are many possible surgical treatments for shoulder instability, but not all are appropriate in patients with hEDS and posterior instability. Procedures like capsulorrhaphy^49^ and Bankart repairs,^10^ which rely on soft tissue healing to stabilize the glenohumeral joint, are associated with a high risk of failure for patients with hEDS.^12^^,^^41^ Conversely, primarily bony restoration procedures, like glenoid osteotomy or a bone block for dysplasia, would fail to address the root cause of the instability—the incompetent soft tissue.^12^^,^^41^ Capsulorrhaphy with allograft augmentation has been proposed to improve joint stability by incorporating normal allograft or autograft collagen into the affected joint.^12^^,^^41^ When 5 cases of shoulder instability secondary to EDS were treated with an Achilles tendon allograft, 4 shoulders experienced improved stability and pain.^41^ It should be noted that these 5 cases were treated for anterior shoulder instability, but this demonstrates the effectiveness of using a tendon allograft for improving shoulder stability in patients with hEDS. Arthroscopic allograft augmentation of the posterior capsule has also been described for patients without hEDS.^29^

There are many well-described successful uses of muscle tendon transfers around the shoulder for pathologies, including subscapularis and posterosuperior rotator cuff tears, brachial plexus injuries, and deltoid deficiency.^23^^,^^25^^,^^26^^,^^39^^,^^54^^,^^57^ Although the LD spans much of the posterior rib cage, it plays an integral role in internal rotation of the shoulder and inserts on the floor of the bicipital groove.^35^ Contraction of the LD, acting with the teres major and pectoralis major, causes arm adduction and internal rotation, while also providing a posterior force on the humeral head.^35^ When the balance between internal and external rotators is disrupted, as in during an electric shock, seizure, or active spasms, posterior dislocation can occur.^17^ Increased activation of the LD, as measured by electromyography, is associated with posterior shoulder instability,^34^ contrasted to the pectoralis major’s association with anterior shoulder dislocation.^7^ As seen in patients with posterosuperior rotator cuff pathology, the LD tendon can be transferred to the GT, where it becomes an external rotator and anterior directed force.^6^^,^^13^^,^^18^^,^^21^^,^^30^^,^^31^^,^^43^^,^^54^^,^^56^ While release of the LD can be considered the most important step in treating shoulder instability, transfer of the LD to the GT adds an extra augmentation of dynamically rebalancing the shoulder to provide an anterior force in a shoulder with a posterior predominance instability pattern. Thus, we suspect an LD release alone would not be enough given the significant capsular laxity with the posterior soft tissue envelope.

Patients with hEDS experience various forms of scapular dyskinesia, driven by PM overactivity and voluntary or involuntary contractions of other periscapular muscles.^52^^,^^53^ The scapular dyskinesia usually begins as the body’s attempt to compensate for the shoulder instability, but only serves to worsen the instability itself.^12^^,^^44^ For example, scapular protraction and dyskinesia during attempted motion can lead to what appears like posterior glenohumeral instability. Many patients with scapular pathologies are actually misdiagnosed and treated for glenohumeral pathologies without recognizing the scapular pathologies driving them.^1^^,^^12^^,^^24^^,^^45^^,^^46^^,^^51^ Thus, as in this case, it is important to evaluate and treat scapular dyskinesia in the setting of complex shoulder instability, such as those with hEDS.

## Conclusion

As described in this case, the arthroscopically assisted posterior transfer of the LD tendon to the lateral GT turns it into an external rotator, providing improved shoulder stability through an anterior-directed force. This dynamic solution to shoulder instability is similar to the concept of the Latarjet for anterior shoulder instability, where the coracoid process is transferred to the glenoid.^9^^,^^27^ Furthermore, the release of the PM helps to correct the scapular dyskinesia driving the posterior instability. We present the use of the arthroscopic-assisted LD tendon transfer and PM release for recurrent posterior instability in hEDS as a novel consideration for this challenging pathology.

## Disclaimers:

Funding: No funding was disclosed by the authors.

Conflicts of interest: Michael B. Gottschalk receives research support from 10.13039/100008894Stryker Corporation, Konica Minolta, and Arthrex. Eric R. Wagner is a consultant for Stryker Corporation, Zimmer Biomet, Acumed, and OsteoRemedies. He also receives institutional research support from Konica Minolta. The other authors, their immediate families, and any research foundation with which they are affiliated have not received any financial payments or other benefits from any commercial entity related to the subject of this article. Each author certifies that all investigations were conducted in conformity with ethics principles of research.

Patient consent: The patient in this case was informed on the content of and utilization of this report. The patient was informed that confidentiality would be maintained, and the patient provided informed consent for this information to be published.

## Supplementary Data

Video 1 Digital dynamic radiography (DDR) of postoperative range of motion on the patient’s affected shoulder and baseline range of motion on the contralateral side. The affected side shows improved shoulder biomechanics. The contralateral side shows limited range of motion due to posterior subluxation associated with glenoid dysplasia and retroversion
